# Memory Visualization-Based Malware Detection Technique

**DOI:** 10.3390/s22197611

**Published:** 2022-10-08

**Authors:** Syed Shakir Hameed Shah, Norziana Jamil, Atta ur Rehman Khan

**Affiliations:** 1 Institute of Energy Infrastructure, College of Computing and Informatics, Universiti Tenaga Nasional, Kajang 43000, Malaysia; 2 College of Engineering and IT, Ajman University, Ajman 346, United Arab Emirates

**Keywords:** malware analysis, polymorphic malware, memory analysis, machine learning, denoising filters, wavelet transform, computer vision, advanced persistent threat, energy security

## Abstract

Advanced Persistent Threat is an attack campaign in which an intruder or team of intruders establishes a long-term presence on a network to mine sensitive data, which becomes more dangerous when combined with polymorphic malware. This type of malware is not only undetectable, but it also generates multiple variants of the same type of malware in the network and remains in the system’s main memory to avoid detection. Few researchers employ a visualization approach based on a computer’s memory to detect and classify various classes of malware. However, a preprocessing step of denoising the malware images was not considered, which results in an overfitting problem and prevents us from perfectly generalizing a model. In this paper, we introduce a new data engineering approach comprising two main stages: Denoising and Re-Dimensioning. The first aims at reducing or ideally removing the noise in the malware’s memory-based dump files’ transformed images. The latter further processes the cleaned image by compressing them to reduce their dimensionality. This is to avoid the overfitting issue and lower the variance, computing cost, and memory utilization. We then built our machine learning model that implements the new data engineering approach and the result shows that the performance metrics of 97.82% for accuracy, 97.66% for precision, 97.25% for recall, and 97.57% for f1-score are obtained. Our new data engineering approach and machine learning model outperform existing solutions by 0.83% accuracy, 0.30% precision, 1.67% recall, and 1.25% f1-score. In addition to that, the computational time and memory usage have also reduced significantly.

## 1. Introduction

Cybersecurity is the protection of internet-connected devices and services from malicious attacks by hackers, spammers, and cybercriminals. As billions of network devices connect and share potentially sensitive data, there is a need for greater security [1]. The practice is used by companies to protect against phishing schemes, ransomware attacks, identity theft, data breaches, and financial losses. To launch the attack, these cybercriminals use malicious files, often called malware. Malware is malicious software that is designed with the specific intention of causing disruption or leakage of information. There are many types of malware, such as spyware, viruses, bots, trojans, file-less malware, adware, ransomware, worms, rootkits, keyloggers, and many more [2]. Malware can be categorized into three types, including polymorphic, metamorphic, and obfuscation [3]. In polymorphic, the malicious file continuously modifies its identifying characteristics to evade detection. One component of polymorphic malware is constantly evolving, while the other remains constant. This type of malware is difficult to detect using conventional methods such as signature-based detection. It becomes extremely dangerous when an APT actor uses polymorphic malware that resides in the system’s main memory to avoid detection.

Malware has increased exponentially through the daily creation of new malware and variants of existing malware. According to a report [4], every day, 338,860 new malware and potentially unwanted applications (PUA) are created. Similarly, another report [5] stated that 450,000 new malware and PUA are created every day. These malicious malware target a variety of devices, including mobile phones and computers. The study shows mobile malware attacks also significantly increased in recent years [6]. Mobile devices have detected 6,463,414 instances of malware, adware, and riskware, according to Kaspersky’s first-quarter report [7]. These malicious programs are either associated with mobile banking trojans or ransomware trojans. According to a report [8], a total of 1,216,350,437 attacks on Personal Computers (PC) are detected from online resources around the world. The statistics shows that Microsoft Windows operating system is the easiest target for malware developers [5].

### 1.1. Background

To develop a detection mechanism, it is vital to understand the malware behaviors that determine the intention of the malware developer. Much research conducted in the past analyzed suspicious files and extracted important artifacts that perform distrustful activities. The process of understanding malware behavior is called malware analysis [9]. There are different analysis techniques called static, dynamic, hybrid, and memory forensics/analysis [10]. In static analysis, it is not necessary to run the code to perform the analysis [11]. Instead, the static analysis looks through the file to see if it contains any indicators of malicious intent. Identifying malicious infrastructure, libraries, or packed files can be facilitated by using this technique. File names, hashes, strings, IP addresses, domain names, and information contained within file headers are some of the technical indicators that can be used to determine whether or not a file contains malicious content. In addition, tools such as disassemblers and network analyzers may be used to examine malware without executing it to gain information about its activity. Since static analysis does not execute the code, complex malware with dangerous runtime behavior might elude detection [12,13]. For instance, if a file emits a dynamic string that downloads a malicious file, it would be undetectable by simple static analysis. To get a better understanding of the behavior of a file, businesses have turned to dynamic analysis.

In a sandbox, suspicious harmful code is executed in a protected environment [14]. This closed system enables security experts to monitor the activity of malware without the danger of infection or egress into the company network. The dynamic analysis gives greater visibility to threat hunters and incident responders, allowing them to assess the exact nature of a threat. Automated sandboxing reduces the time necessary to reverse-engineer a file to find harmful code. The challenge with dynamic analysis is that opponents are smart and know of the existence of sandboxes [15]; therefore, they are good at spotting them. To fool a sandbox, opponents hide latent code into their bodies.

Basic static analysis is not a reliable way for identifying advanced malicious code and may sometimes circumvent sandbox protection. By integrating static and dynamic analysis, the hybrid analysis offers security teams the advantages of both techniques [10]. It may first identify malicious code that is trying to obscure itself and then extract a large number of indicators of compromise (IOCs) through dynamic analysis. Hybrid analysis facilitates the detection of unknown threats, particularly those provided by the most sophisticated malware. Hybrid analysis, for example, uses static analysis to data provided by behavioral analysis, such as when malicious code runs and causes memory modifications.

Memory malware analysis is commonly employed for digital investigation and malware analysis. It refers to the process of analyzing a dumped memory image from a targeted system after malware execution to obtain a variety of artifacts [16], such as network information, running processes, API hooks, kernel-loaded modules, and Bash history, etc. This phase is crucial because it is always advisable to have a better grasp of the malware’s capabilities. Volatility tools are frequently employed to extract relevant artifacts from memory images. Volatility is an open-source memory forensics framework designed for incident response and malware examination. This is a very powerful tool that can interact with memory dump files in numerous ways. However, it requires domain expertise to extract relevant artifacts from the malware memory dump and also takes a considerable amount of time. Therefore, researchers proposed several visualization-based techniques for detecting and categorizing malware classes.

### 1.2. Problem Statement

Numerous researchers have proposed a visualization-based detection technique capable of analyzing the behavior of malware to overcome the obstacles posed by other detection techniques. Visualizing malware as a color or in grayscale image provides the advantage of differentiating different components of the malware; however, it is crucial to use appropriate artifact extraction techniques for the malware images. Numerous techniques are employed, including global image descriptor (GIST), histogram of oriented gradient (HOG), principal component analysis (PCA), Kernel PCA, and many others. However, these feature-engineering techniques are either too complicated or require a great deal of computing and memory power. To the best of our knowledge, no researchers have removed noise added to malware images during the transformation and coding process. These noises are artifacts produced by random pixel values in an image that does not originate from the source, and they have a direct impact on the performance [17]. Hence, it is a need to understand, investigate, and develop an efficient technique that can remove the noise from the malware image.

### 1.3. Objectives and Contribution

In this study, we convert all malicious and benign memory dump files into 224 × 224 pixels of grayscale images. In image processing, noise reduction is one of the most crucial steps [18,19]. These noises may be introduced during the transformation [20] from computer memory to dump files or from dump files to grayscale images. We propose the Non-Linear Means denoising technique for removing noise from images to restore the original state. Since noise, edge, and texture are high-frequency components, it is difficult to distinguish them during the denoising process, and denoised images may lose detail. The second important step is to compress the denoised images of malware to reduce the irrelevance and redundancy of the data. Image compression serves primarily to reduce the number of bits required to represent an image. Finally, the compressed images are fed into machine learning classifiers for testing and training in order to detect and classify various malware classes.

We make the following contributions in this paper,

- We examine various image noises, including Gaussian, Salt Pepper, Poisson, and Speckle, and apply various filtering techniques to minimize the distortion in these images.
- We present a technique using discrete wavelet transform to compress the malware images to reduce the irrelevant and redundant data in the images, without compromising important information.
- We present various machine learning classifiers and experiments with their appropriate hyperparameters to tune them to obtain a significant result.

### 1.4. Paper Structure

The structure of the subsequent sections is as follows: Section 2 describes the relevant work. In Section 3, a thorough summary of the methodology is presented. Section 4 displays the experiment, result, and findings. Section 5 delivers the discussion, and Section 6 provides the conclusion.

## 2. Related Work

Researchers employed a variety of methodologies to investigate and identify malware. A number of researchers employed static analysis in their hunt for malicious software [21,22,23,24,25,26,27]. However, due to limitations, such as vulnerability to unseen malware, time consumption, and manual feature extraction, etc., it is not feasible to use. Apart from static analysis, dynamic malware analysis is also widely used by various researchers [28,29,30,31,32]. Similarly, researchers also proposed the integration of static and dynamic analysis approaches for malware detection and classification [9,33,34,35]. However, these analysis techniques also have some limitations and, as of today, the most widely used approach for malware analysis is memory analysis.

In [36], researchers present a memory forensics-based malware detection technique to detect and classify various malware classes. The authors transformed the memory dump file into grayscale images. Two important techniques—contrast limited adaptive histogram equalization and wavelet transform of level two—are used to remove the noise and compress the image. Various machine learning classifiers are used, and SVM with RBF kernel obtained the best result, with an accuracy of 97.01%, precision of 97.36%, recall of 95.65%, and f1-score of 96.36%.

In [16], researchers present malware detection based on an integrated approach of memory forensics and dynamic analysis. The suspicious characteristics are extracted using memory and dynamic analysis. The combined feature characteristics are fed into the machine learning classifiers, such as support vector machine (SVM), decision tree (DT), naïve bayes (NB), k-nearest neighbor (KNN), and random forest (RF). The SVM outperformed other classifiers, with an accuracy of 98.50% and a false positive rate (FPR) of 1.7%, respectively.

In [37], researchers present a malware detection and classification approach based on memory analysis. In their approach, authors extract features, such as API calls, DLLs, process information, hidden code injection, and communication log, from the malware memory images. A feature selection technique such as information gain (IG) and correlation is used to select the most relevant artifacts. Finally, the highest weight of artifacts is fed into the machine learning classifiers, such as NP, SVM, KNN, DT, and RF. Among all other classifiers, SVM obtained the highest accuracy of 98.5% and a false positive rate of 1.24%.

In [38], researchers present malware detection and classification based on the malware’s memory dump. The memory dump files are converted into grayscale images with a fixed size and dimensions. A histogram of oriented gradient (HOG) is used to extract the vital artifacts from grayscale images. Various classifiers, such as SPAM-GIST, HPC, KNN, RF, and multilayer perceptron (MLP), are used. Among all, an MLP obtained the highest accuracy of 95.2%, precision of 95.10%, recall of 93.20%, and f1-score of 94.10%.

In [13], researchers present malware detection and classification based on memory forensics, manifold, and computer vision. Authors convert the memory dump files into color images that consist of three channels r (red), g (green), and b (blue) with various dimension sizes such as 224 pixels, 300 pixels, 4096 pixels, and a square root scheme. Two important feature extraction techniques, HOG and global image descriptor (GIST), are used to extract the most relevant features from the color images. The combined features of HOG and GIST are later fed into machine learning classifiers such as RF, SVM (RBF, Linear), XGBoost, and J48. Among all other classifiers, SVM with RBF kernel obtained the highest accuracy of 96.36%, precision of 96.40%, recall of 96.40%, and f1-score of 96.40% for a classification problem.

In [39], researchers describe how memory dump files can be used as a heuristic environment for detecting malware. In their approach, the authors collect features such as registry activities, import libraries, and API function calls from the memory dump files. Before feeding the extracted features into the machine learning classifiers, they evaluate each feature in terms of significance values. The SVM classifier achieved the best accuracy of 96%.

In [40], researchers present malware detection techniques based on memory dumps and static analysis. The authors claim that, using their approach, one can reveal the hidden characteristics of suspicious files. The authors first used static analysis for packed malware samples and obtained 90% of the data. In addition, they claim that their approach can be used in a variety of strategies to improve the malware detection rate.

In [41], researchers present a novel solution using trigger-based memory analysis. With this approach, memory dumps are taken place when a specific or desired event occurs. There are many problems such as user-mode and kernel-mode root kits, and calling native functions that are not hooked, which can be treated with the approach claimed by the authors. Combining this approach with other memory analysis can improve security and provide important information for malware detection.

In [42], researchers present malware detection based on trustworthy volatile memory dumps from a virtual machine in the private cloud of the organization. The authors used the MinHash technique to examine the data in the dump files. This approach is further accessed in terms of efficacy by using various machine learning classifiers and similarity-based techniques. The authors claim their proposed framework can identify the known and unseen suspicious files with a high true positive rate (TPR) and low false positive rate. Their framework is tested and obtained a TPR of 100% and FPR of 1.8% for the ransomware and RATS, respectively.

In [43], researchers present an agentless sandbox solution independent of the virtual machine’s (VM) hypervisor for malware detection. Using the VM’s introspection method, it kept an eye on malware running in memory data outside of the VM and analyzed its system behaviors, such as process, file, registry, and network activities. The authors used 20 advanced and eight script-based malware samples to test their approach and determine the viability. The results show that their approach could be used to analyze suspicious malware activities that are helpful in cyber security.

In [44], researchers present a visualization method of malware detection using deep learning. In this approach, in Cuckoo, the API trigger is replaced with hooking, which indicates that API calls and memory dumps are synchronized. This is due to the encrypted IP address being stored in the memory. The authors claim that it is feasible to retrieve the plain text and identify the hidden information before malware hides it again.

In [45], researchers present a novel approach to capturing a memory from a system infected by ransomware malware such as NotPetya, Bad Rabbit, and Phobos hybrid. The contents of the memory are examined with live forensic tools to determine the symmetric encryption keys in use. Two critical steps are used to recover the AES keys. First, a timeline is manually created by combining data from multiple sources to show ransomware’s behavior and when encryption keys are in the memory and for how long. Second, encrypted files are decrypted using the found keys. The authors stated that the generated timelines are an excellent way to visualize ransomware behavior and encryption key management practices from a forensic investigation and mitigation perspective when the encryption keys are in the memory.

In [46], researchers propose a VMI, MFA, and hypervisor machine learning combination to construct an upgraded VMM-based A-Introspection Extraction (A-InExt) system. They employ VMI to examine digital artifacts from a live guest operating system to comprehend the processes. A-IntExt features an intelligent cross-view analyzer (ICVA) that searches VMI data for hidden, dead, and suspicious processes, while predicting the early execution of malware on the guest OS. Malicious MFA executables are discovered by methods using machine learning. The A-IntExt system is put to the test by running malicious and benign executables on the live guest OS. It identifies unknown malware on a produced dataset with 99.55% accuracy and a 0.004% false positive rate. A-IntExt outperformed VMM’s detection of malware by 6.3%.

In [47], researchers present signature matching and string pattern matching criteria for the YARA scanner to identify malware in RAM dumps. To reduce time, a GUI-based automated forensics toolkit is employed for process analysis rather than the command-line method of the volatile memory forensic toolkit. A built-in software examines the RAM image to discover and uninstall potentially harmful applications. According to the research, malware forensics may be used in the recorded processes to analyze the attack’s sources and identify the root cause.

## 3. Methodology

**Figure 1:**
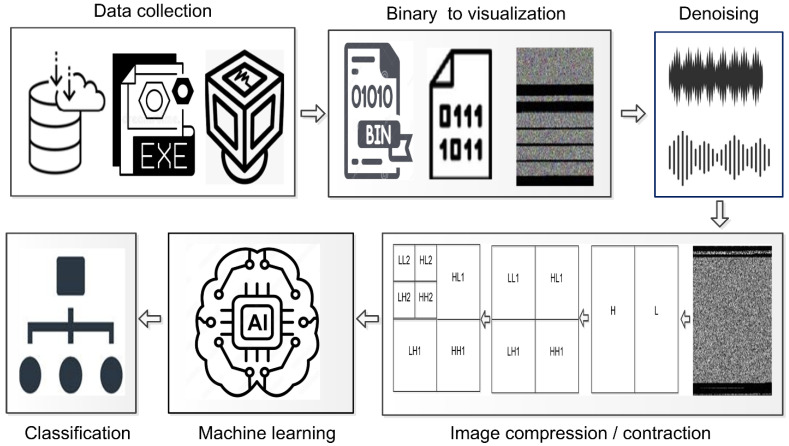
Proposed methodology.

The methodology that we propose includes the following five primary steps: (i) data collection; (ii) binary to visualization; (iii) denoising; (iv) image compression/contraction; (v) machine learning classifiers; and (vi) classification. The step of data collection is comprised of a couple of sub-steps, such as the execution of portable executables (PE) within a virtual box. This is to collect an image of the system’s random-access memory (RAM), which contains all malicious activities. In some of the literature, these memory-based images are also referred to as memory dump files. Essentially, memory dump files are binary files. In the second step, these binary files are converted to grayscale images for visualization purposes. Next, we experiment with a number of noise reduction techniques, including contrast limited adaptive histogram equalization (CLAHE), non-local means denoising filter, bilateral filter, denoise total variation (TV), Chambolle filter, and Wiener filter. A discrete wavelet transformer (DWT) technique is used to compress and reduce the size of grayscale malware images without losing many important details. The output images from the preceding step are then fed into machine learning classifiers. The proposed methodology is shown in Figure 1.

### 3.1. Data Collection

Data play a crucial role in the research process. Therefore, it is essential to collect such information from a trustworthy source. To understand what a dataset is, it is necessary to examine its components. It can be in various formats, including text, audio, and video. In the area of malware detection and classification, four large datasets are often used to find and categorize malware. These datasets are BIG2015 [48], MalImg [49], Malicia [50], and Malevis [51]. However, none of these datasets contains the PE files to examine the source file. Therefore, we use a memory forensics-based malware dataset [52] that contains a total of 4294 malware and benign dump files. These dump files are obtained through the creation of a virtual Windows 10 environment. The system’s memory image is extracted using Prodcump and saved as a dump file. Table 1 shows a breakdown of the proposed dataset used in this paper.

- Adware

Adware is software that is supported by advertising and displays advertisements on devices such as computers and mobile phones. Adware can also be referred to as madware when it is installed on a mobile device [53]. Adware targets both consumers and businesses. However, it primarily targets individuals, typically by luring them in with the promise of new content (such as games, movies, or deals).

- Worm

A worm is a type of malicious software that can make copies of itself and spread throughout a network by taking advantage of holes in the system’s security. Attachments sent via email, text messages, file-sharing programs, social networking sites, network shares, removable drives, and software vulnerabilities are all potential vectors for its propagation.

- Trojan

Trojans are deceitful programs that give the appearance of carrying out one function, but, in reality, carry out another malicious function. They may pose as free software, videos, or music, or even as advertisements that look completely legitimate.

- Virus

A computer program can replicate itself and spread to another computer without the user’s knowledge or permission. If a computer is infected with a virus, the information stored on it could be erased or corrupted.

### 3.2. Binary to Visualization

A file without any text is referred to as a binary file. It stores information as bytes, which are typically interpreted differently than characters in text files. Its primary function is to simplify data storage. Typically, the headers of these files contain instructions on how to access the data it contains. These can store any type of data required by a computer system. The memory dump files obtained in the preceding step are referred to as binary files. In this step, these binary files are turned into color images with different sizes depending on how big they are. We use a python script to do the conversion process from binary into color images [54]. The transformation of the binary to visualization process takes two important steps: (i) binary to 8-bits conversion and (ii) 8-bits to image presentation.

**Figure 2:**
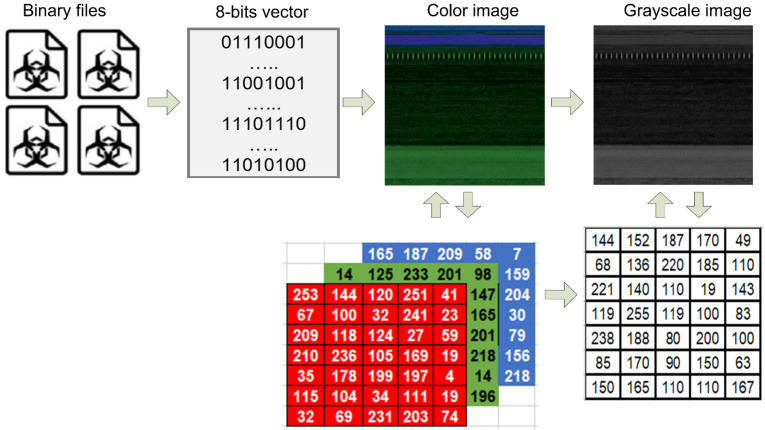
The transformation process from binary to grayscale image.

The term “8-bit” refers to a method of storing graphic information in the memory of a computer or an image file, with each pixel represented by 8 bits (1 byte). These images are capable of being rendered in color or grayscale. Grayscale images only have a single channel, whereas color images have three: R (red), G (green), and B (blue). The pixel range of these images is between 0 and 255 and it is calculated through 2^n^ − 1, where n is the bit number. These images are the combination of pixels that build a digital image. A pixel is one of the many tiny dots or squares that combine to form an image on a computer screen. A greater number of pixels makes an image appear more realistic or precise. These pixel values specify how dark or light the images are. Each pixel value in an image represents a significant aspect of the image. The graphical process of the binary to visualization process is depicted in Figure 2.

As previously stated, the size of malicious image files is dependent on the size of the binary file. The visualization process produces images with a dimension range of 1024 to 1580 pixels. Initially, we transform the binary files into color images with dimensionality depending on the size of the individual file in order not to lose any information in different color channels (RGB). The information in Table 2 illustrates how to determine the dimensions of each image. Algorithm 1 illustrates how the malware binary files are initially transformed into color images in ‘PNG’ format.

**Figure 3:**
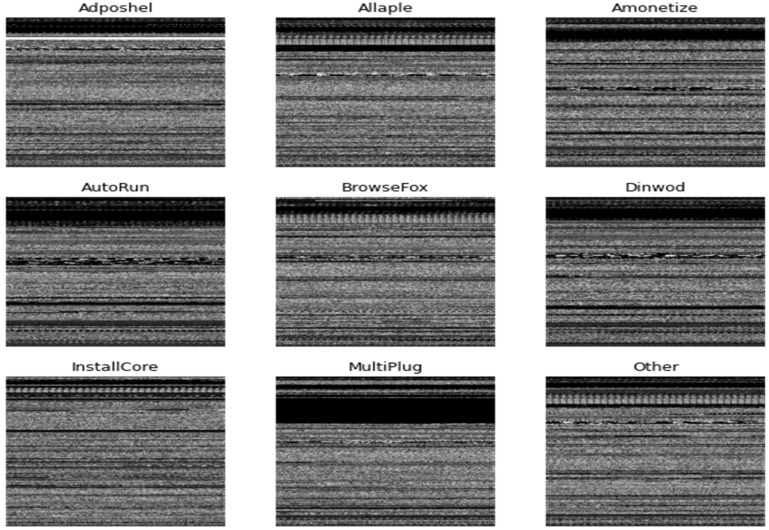
Grayscale images of various malware classes after the transformation process.

Due to its high dimensionality, it necessitates an enormous amount of storage and processing power. We propose a fixed size of 224 × 224 pixels for all images to reduce memory consumption and computational expense using a computer vision library in Python. Figure 3 depicts the grayscale images of various classes of malware.

**Table 2:** Determination of Image dimensionality.

S. No	File Range	Dimensions
1	File_size < 10,240	32
2	10,240 <= File_size <= 10,240 * 3	64
3	10,240 * 3 <= File_size <= 10,240 * 6	128
4	10,240 * 6 <= File_size <= 10,240 * 10	256
5	10,240 * 10 <= File_size <= 10,240 * 20	384
6	10,240 * 20 <= File_size <= 10,240 * 50	512
7	10,240 * 50 <= File_size <= 10,240 * 100	786
8	Else	1024

**Algorithm 1:** Transformation algorithm for dumping (binary) files to RGB images
 Input: A path to a dataset of memory dump files
 Output: Transformation of RGB images with various dimensionality
 Start Set *B* a list of memory dump files Set *width* None Set *height* None Set *data* an empty list [ ] For *i* in length(B) where *i* integer numbers range from 0 to n. If file_size of B[i] < 10,240 *width* = 32 If 10,240 <= file_size of *B[i]* <= 10,240 * 3 *width* = 64 If 10,240 * 3 <= file_size of *B[i]* <= 10,240 * 6  *width* = 128 If 10,240 * 6 <= file_size of *B[i]* <= 10,240 * 10  *width* = 256 If 10,240 * 10 <= file_size of *B[i]* <= 10,240 * 20  *width* = 384 If 10,240 * 20 <= file_size of *B[i]* <= 10,240 * 50  *width* = 512 If 10,240 * 50 <= file_size of *B[i]* <= 10,240 * 100  *width* = 786 Else  *width* = 1024 End If  *height* = (file_size/*width*)/+1 image = Width × *height* image.save[‘PNG’] *data*.append[image] End For End

### 3.3. Denoising

The presence of artifacts in an image that do not originate from the content of the original source itself is referred to as noise in the image [20,55]. In a broad sense, noise can be defined as a statistical variation of a measurement that is produced as a result of a random process. In digital images, noise emerges as an artifact in the image and appears as a grainy structure covering the image. This can be seen when comparing two images side by side. Noise is an unwelcome or disturbing artifact that, in most cases, lowers the subjective quality of an image. Noise can manifest itself in a number of distinct ways and can take on a variety of appearances within an image. It is a challenging task to remove noise from an image because the noise is linked to the high-frequency content, also known as the details. As a consequence of this, the objective is to find a solution that minimizes the amount of background noise, while minimizing the amount of information that is lost.

The presence of noise in an image can have either an additive or multiplicative impact on the image’s overall quality, depending on the nature of the noise. In the Additive Noise Model, presented in Equation (1), a corrupted noisy signal is produced by adding an additive noise signal to the original signal. On the other hand, the Multiplicative Noise Model, presented in Equation (2), multiplies the authentic signal by the noise signal:

(1) I(x, y)= o(x,y)+ n(x,y)

(2) I(x, y)= o(x,y)× n(x,y)

where o(x,y) is the intensity of the original image, and n (x,y) is the noise that is added or multiplied to produce the corrupted signal I (x,y) at the (x,y) pixel position.

Noise can be introduced into a digital image by a variety of means, including during the process of acquiring the image, transmitting it, and encoding it. The number of pixels in an image that is damaged can be used to quantify the amount of noise in the image. There is a very broad spectrum of different kinds of noise. We concentrate primarily on the most significant types, which are gaussian noise, salt-and-pepper noise, poisson noise, and speckle noise.

- Gaussian Noise

**Figure 4:**
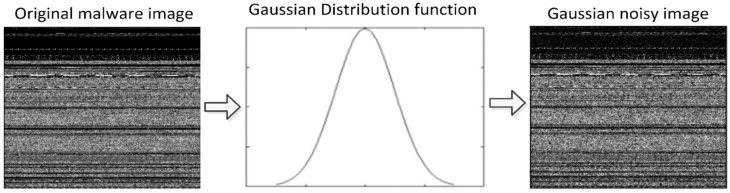
Gaussian noisy image.

Gaussian noise is a well-established type of statistical noise with a probability density function (PDF) that is equivalent to the normal distribution [56]. The presence of Gaussian noise is characterized by a signal-wide distribution that is constant. A noisy image has pixels that are composed of the sum of their original pixel values, as well as a random value based on Gaussian noise. A bell shape can be seen when looking at the probability distribution function for a Gaussian distribution. The most normal deployment of additive white Gaussian noise can be found in a variety of different applications for Gaussian noise. The Gaussian distribution function of Gaussian noise, also known as the probability distribution function, is depicted in Figure 4, along with a pixel representation of Gaussian noise. Equation (3) is used to compute the Gaussian noisy image.

(3) $$p_{G}\left(z\right)=\frac{1}{\sigma \sqrt{2\pi }}e^{-\frac{{\left(z-\mu \right)}^{2}}{2\sigma^{2}}}\;.$$

where z represents a gray level, μ the mean value, and σ the standard deviation.

- Salt-and-Pepper Noise

**Figure 5:**
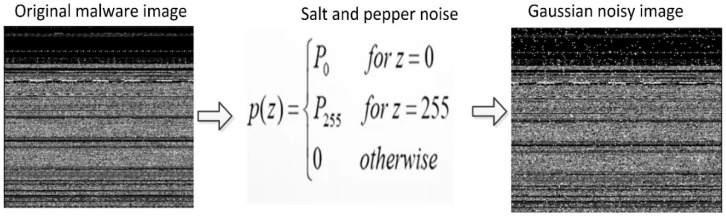
Salt-and-Pepper noisy image.

This type of noise is exclusively present in grayscale images (black and white images). As the name implies, salt (white) in pepper (black) has white spots in the dark regions, while pepper (black) in salt (white) has black spots in the white regions [57]. In other words, an image with salt-and-pepper noise contains a small number of dark pixels in bright regions and a small number of light pixels in dark regions. Salt-and-pepper noise is known as impulse noise as well. It can be brought on by a variety of factors, including dead pixels, analog-to-digital conversion errors, and bit transmission errors, etc. The application of a median filter, morphological filter, or contra harmonic mean filter can effectively eliminate this type of noise, as well as other types of noise. Salt-and-pepper noise is visible whenever there is a sudden change in brightness or contrast in an image. Figure 5 illustrates how the malware image is transformed into a noisy image after adding the salt and pepper.

- Speckle Noise

**Figure 6:**
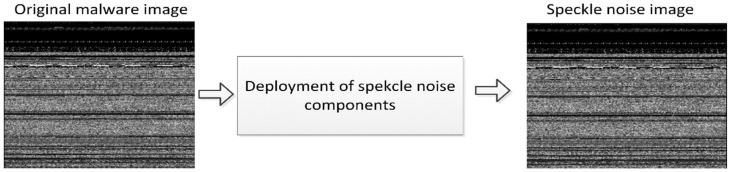
Speckle noisy image.

Speckle noise is a multiplicative type of noise, in contrast to Gaussian noise and salt-and-pepper noise. This causes a reduction in image quality during diagnostic examinations by giving images the appearance of a backscattered wave, which is caused by a large number of microscopic reflections that are dispersed as they flow through internal organs [58]. The observer has a more difficult time differentiating the fine details in the images as a result of this. This particular kind of noise can be seen in the images produced by a wide variety of systems, such as synthetic aperture radar (SAR) and ultrasound imaging, among a great number of others. The speckle noise is presented in Equation (4),

(4) Ob(x,y)= Or(x,y)Ml(x,y)+Ad(x,y)

where Ob, Or, Ml, and Ad represent the observed image, original image, multiplicative component, and an additive component of the speckle noise, respectively. Figure 6 depicts the observed image following the application of speckle noise. It is not easy for a human to differentiate between original malware and a speckle noise image. However, if it is converted into matrix format, you may discover that the pixel values of both images have significantly diverged from one another.

- Poisson Noise

The statistical properties of electromagnetic waves, including X-rays, visible light, and gamma rays, are to be held responsible for the appearance of this noise. Sources of X-rays and gamma rays release a certain number of photons in a given amount of time. In medical imaging systems that use X-rays and gamma rays, these rays are injected into the patient’s body directly from their point of origin. These sources exhibit unpredictable fluctuations in the number of photons. The image that is gathered as a result has randomness in both space and time. This noise is also referred to as quantum noise (photon noise), as well as shot noise. The Poisson noise distribution can be represented in Equation (5),

(5) $$P\left(X=x\right)=\frac{\lambda^{x}e^{-\lambda }}{x!}$$

where, x = 0, 1, 2, 3, 4…,   λ is the number of occurrences in the interval, and e is the Euler’s constant.

**Figure 7:**
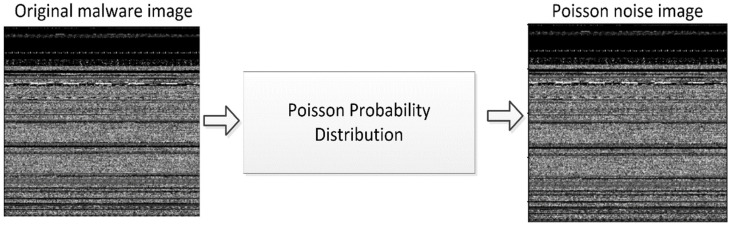
Poisson noisy image.

Figure 7 depicts the malware image after the Poisson Probability Distribution is deployed.

Denoising an image means removing noise from an already noisy image to restore it to its original state. Denoising an image, however, can make it difficult to differentiate between noise, edges, and textures, because these are high-frequency components. As a result, the denoised images may invariably lose some details. In general, one of the most important challenges is to extract meaningful information from images that are contaminated with noise during the process of noise removal to produce images of high quality. However, it is still a difficult and potentially open-ended task. The primary reason for this is that, from a mathematical point of view, image denoising is an inverse problem, and its solution is not unique. This means that there is more than one way to solve the problem; therefore, it is required to use various filter techniques to minimize the noise from images. The term “noise filtering” refers to a series of processes that are carried out to eliminate the noise that is associated with the image data that are obtained from construction and infrastructure sites. As we do not know what type of noise is present in the malware images, it is necessary to employ an efficient and effective denoising technique to compensate for the data corruption that has occurred. The use of spatial filters such as means and medians is employed to get rid of image noise. Despite this, spatial filters have the undesired effect of blurring the edges of images, in addition to smoothing the data to reduce noise. In our paper, we propose the various denoising filter techniques listed below:

- Contrast Limited Adaptive Histogram Equalization (CLAHE)

To improve image contrast, we use contrast limited adaptive histogram equalization (AHE). Ordinary histogram equalization computes a global equalization, whereas adaptive histogram equalization computes multiple histograms, each corresponding to a distinct section of the image, and uses them to redistribute the image’s lightness values. It is appropriate for enhancing local contrast and defining edges in each image region. CLAHE, a variant of adaptive histogram equalization, limits AHE’s tendency to overamplify noise in relatively homogeneous image regions. It is capable of performing histogram equalization in small patches or small tiles, while maintaining a high level of accuracy and contrast limiting. This small region is referred to as tiles. The adjacent tiles are then merged using bilinear interpolation to eliminate the artificial borders. This algorithm can be utilized to enhance image contrast.

- Bilateral Filter

When smoothing with a Gaussian distribution, we take the weighted average of the surrounding pixel values. The weights are calculated in inverse proportion to the distance from the geographic center of the neighborhood. The bilateral filter adds a tonal weight, in addition to these spatial weights. Pixel values that are relatively similar to the pixel value in the center are weighted more heavily than more dissimilar pixels. Due to the tonal weighting, the bilateral filter can preserve edges, which are defined as significant tonal value differences, while smoothing out relatively flat regions. The bilateral filter image can be defined in Equation (6) and Equation (7),

(6) $$I^{\mathrm{SBF}}\left(y\right)=\frac{1}{K^{q}}\sum_{y_{i}\in \Omega }I\left(y_{i}\right)h_{\sigma_{r}}\left(\|I\left(y_{i}\right)-I\left(y)\right\|\right)h_{\sigma_{s}}\left(\left\|y_{i}-y\right\|\right)\;$$

and,

(7) $$K^{q}=\sum_{y_{i}\in \Omega }h_{\sigma_{r}}\left(\|I\left(y_{i}\right)-I\left(y)\right\|\right)h_{\sigma_{s}}\left(\left\|y_{i}-y\right\|\right)$$

where $I^{\mathrm{SBF}}$ represents the denoised image, I is the actual image, Y represents the coordinates of pixels that are required to be filtered, Ω represents window that are centered pixels, $h_{\sigma_{r}}$ represents the Gaussian range kernel, and $h_{\sigma_{s}}$ represents the space kernel.

- Wiener Filter

When an image is blurred by a known lowpass filter, it is possible to recover the image using inverse or generalized inverse filtering, which is a deconvolution restoration technique. In other words, it is possible to recover an image after it is blurred by a filter. If we take the approach of reducing one type of degradation at a time, we can develop a restoration algorithm for every type of degradation, and then easily combine these algorithms. By executing a tradeoff, the Wiener filtering method achieves the optimal balance between inverse filtering and noise smoothing. It eliminates the additive noise, while simultaneously inverting the blurring. Regarding the mean square error, Wiener filtering achieves the best results possible. In other words, it aids in reducing the overall mean square error and smoothing out the noise during the inverse filtering process. The Wiener filtering technique produces a linear approximation of the original image. It can be presented in Equation (8)

(8) $$W\left(f_{1},f_{2}\right)=\frac{H^{*}\left(f_{1},f_{2}\right)S_{\mathrm{xx}}\left(f_{1},f_{2}\right)}{{\left|H\left(f_{1},f_{2}\right)\right|}^{2}S_{\mathrm{xx}}\left(f_{1},f_{2}\right)+S_{\eta \eta }\left(f_{1},f_{2}\right)}$$

where $f_{1},f_{2}$ are the pixel values at the x-axis and y-axis, $S_{\mathrm{xx}}\left(f_{1},f_{2}\right)$ is the power spectrum of the original image, $S_{\eta \eta }\left(f_{1},f_{2}\right)$ is the power spectrum of the additive noise, and $H\left(f_{1},f_{2}\right)\;$ is the blurring filter. The Wiener filter has two distinct components, an inverse filtering component and a noise smoothing component. Not only does it perform deconvolution by inverse filtering (high-pass filtering), but it also eliminates noise with a compression operation (low-pass filtering).

- Gaussian Filter

An image is processed with a low-pass filter known as a Gaussian filter to reduce noise (high-frequency components) and blur specific areas of the image. To achieve the desired result, the filter is implemented as an Odd-sized Symmetric Kernel (DIP version of a Matrix) that is applied to each pixel of the Region of Interest (RoI). The kernel is insensitive to abrupt color changes (edges) because the central pixels contribute more to the final value than those on the periphery. The Gaussian filter is a Gaussian function approximation. To apply a Gaussian filter to an image, we must first specify the size of the kernel/matrix used for denoising the image. The sizes are typically even numbers, allowing the central pixel to compute the overall results. In addition, kernels are symmetric and have the same number of rows and columns. The Gaussian function computes the values within the kernel, as shown in Equation (9),

(9) $$G\left(x,y\right)=\frac{1}{2{\pi \sigma }^{2}}e^{-\frac{x^{2}+y^{2}}{2\sigma^{2}}}$$

where x and y are two image coordinate values, π the constant PI value 3.13, and σ represents the standard deviation

- Total variation

Total variation (TV) denoising is a noise removal process that is used in signal processing, more specifically, in image processing. It is also known as total variation regularization and total variation filtering (filter). It is predicated on the idea that signals that contain an excessive amount of information, some of which may be dubious, have a high total variation; more specifically, the integral of the absolute image gradient is high. According to this principle, removing unwanted detail, while maintaining important details such as edges, can be accomplished by lowering the total variation of the signal, provided that the reduced signal is an accurate representation of the original signal. This method of removing noise has advantages over more straightforward techniques such as linear smoothing and median filtering, both of which reduce noise but also smooth away edges to a greater or lesser degree. Total variation denoising, on the other hand, is an exceptionally efficient edge-preserving filter. This means that it can simultaneously preserve edges, while simultaneously smoothing away noise in flat regions, and it can do this even at low signal-to-noise ratios. In this paper, we use the Chambolle technique of TV in the images to reduce the number of unwanted artifacts from malware images.

- Non-Local Means Denoising

A non-local means of denoising is another technique used to remove noise from images. When smoothing an image, “local mean” filters take the mean value of a group of pixels surrounding a target pixel and use that to determine the smoothness of the image. Non-local means filtering, on the other hand, takes the mean of all pixels in the image and weights it according to how similar each pixel is to the target pixel. In comparison to algorithms based on the local mean, this yields an image that retains much more of its original clarity after filtering, while suffering far less loss of information. As a consequence of this, the method can properly preserve textures that, if processed by another denoising technique, would become blurry. Equation (10) presents a mathematical representation of the non-local means denoising technique where p and q are two points in the image.

(10) $$u\left(p\right)=\frac{1}{C\left(p\right)}\int_{\Omega }v\left(q\right)f\left(p,q\right)\mathrm{dq}$$

u(p)= indicate a filter value at the pixel point p

v(q)= an unfiltered value at point q

f(p,q) = represents a weighted function and C(p) calculates the normalization factor

(11) $$C\left(p\right)=\int_{\Omega }f\left(p,q\right)\mathrm{dq}$$

The objective of the weighting function is to establish the degree to which the image located at point p is connected to the image located at point q. Equation (11) presents a mathematical form to calculate the weighted function where h and B(p) are filter parameters and local mean values, respectively.

(12) $$F\left(p,q\right)=e^{-\frac{|B\left(q\right)-B\left(p\right){|}^{2}}{h^{2}}}$$

Algorithm 2 illustrates how the malware images can be transformed into an image that has the least noise in it.
**Algorithm 2:** Transformation algorithm for the grayscale image to remove noise
 Input: A path to a dataset image
 Output: A set of denoised images
 Start Set data an empty list [ ] For *I* in image data where *i* integer numbers range from 0 to n. For each pixel of a single image Calculate Normalization function (11) Evaluate weighted function Equation (12) Calculate non-local means Equation (10) data.append[denoised image] End For End For End

### 3.4. Image Compression/Contraction

Image compression is a process that is applied to a graphics file to reduce the amount of space that the file takes up in bytes, while maintaining an acceptable level of image quality. If the file size is decreased, then a given amount of space on the hard disk or in the memory can hold a greater number of images. Before beginning the processing of larger images or videos, it is necessary to first compress any images that need to be transferred. An encoder is what accomplishes the process of compressing images and then outputs the image in its compressed form. Mathematical transformations are an extremely important part of the process of data compression. There are various widely used techniques, such as fractal, wavelets, chroma sub-sampling, transform, and run-length encoding. Similarly, there are linear-based filters such as PCA, Factor Analysis (FA), Linear Discriminant Analysis (LDA), and Truncated Singular Value Decomposition (SVD). These dimensionality techniques are either sensitive to the scale of the feature, extracting the feature based on assumption, not robust against a non-linear problem, or have a low interpretation of feature issues, and many more. However, in this paper, we propose to use a wavelet approach.

Wavelet transforms are mathematical tools that can be used to analyze data in which features vary across a variety of scales. In the case of signals, features can take the form of slowly varying trends, transients, or frequencies that change over time. Edges and textures are examples of features that can be found in images. The limitations of the Fourier transform were the primary motivation behind the development of wavelet transforms. Equation (13) present the wavelet transform,

(13) $$F\left(a,b\right)=\int_{-\infty }^{\infty }f\left(x\right)\psi_{\left(a,b\right)}^{*}\left(x\right)dx$$

where * is the complex conjugate symbol and ψ represents a function.

Wavelet transforms can be classified into two broad classes: the continuous wavelet transform (CWT) and the discrete wavelet transform (DWT). A formal tool that is used in mathematics to provide an overcomplete representation of a signal is called the CWT. This is achieved by granting the wavelets’ translation and scale parameters the freedom to continuously vary in value. In contrast, the DWT is an implementation of the wavelet transform that uses a discrete set of wavelet scales and translations, while adhering to some predetermined rules. This transform decomposes the signal into a set of mutually orthogonal wavelets, which is the primary distinction between DWT and CWT. In this paper, we propose to use DWT to compress the malware images without losing too many artifacts for machine learning to distinguish between malicious and benign images.

**Figure 8:**
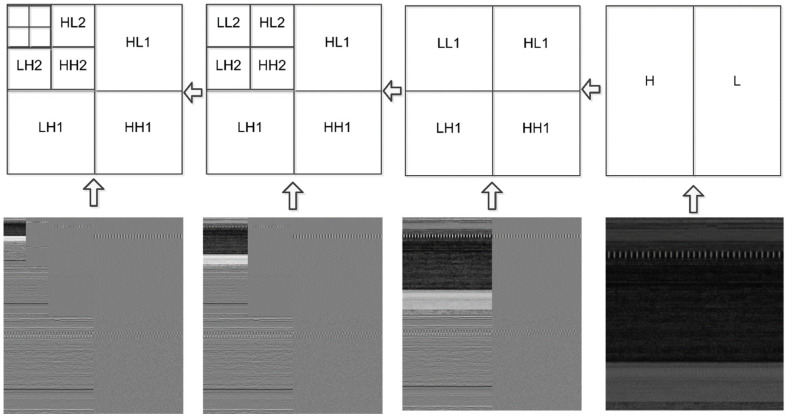
Discrete Wavelet Transform process of level three.

**Figure 9:**
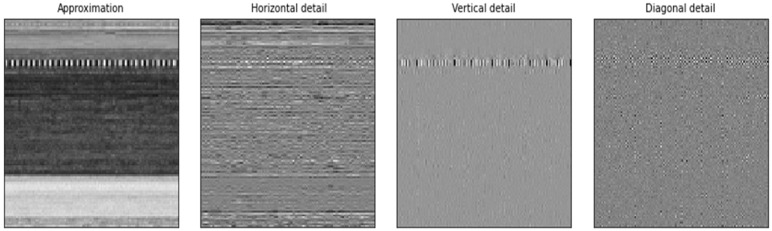
Discrete Wavelet Transformation—single level.

The DWT is most useful when applied to non-stationary signals because of its dynamic nature. By applying this transformation, one can obtain a high temporal resolution for high-frequency components, while maintaining a good frequency resolution for low-frequency components. This method starts with a mother wavelet, which can be a Haar, a Morlet, or a Daubechies, amongst other possible choices. After that, the signal is transformed into scaled and shifted iterations of the mother wavelet. Two filters, high-pass and low-pass filters, are applied to a signal. After that, the image is decomposed into high-frequency (details) and low-frequency components (approximation). At every level, we get four sub-signals. The approximation displays the overall trend of pixel values, as well as the horizontal, vertical, and diagonal details. Figure 8 shows how the malware image is compressed to level three during the DWTC procedure. Figure 9 depicts the transformation process of a single level.

### 3.5. Machine Learning

To automate the process of malware detection and classification, various researchers use machine learning and deep learning. Deep learning algorithms provide many advantages over machine learning classifiers. However, it requires a huge number of data features and high computational costs to train and test it. In this paper, we propose to use machine learning classifiers. The images that are previously compressed during the DWT process are converted into feature vectors without a feature selection process to feed them to machine learning classifiers. Various classifiers are widely used in the research, such as SVM with RBF and Linear kernel, XGBoost, DT, RF, bagging classifier, logistic regression, AdaBoost classifier, gradient boosting classifier, and histogram-based gradient boosting classifier.

### 3.6. Evaluation Metrics

Evaluation is the process of using a variety of evaluation metrics to gain an understanding of the performance of a machine learning model, as well as the strengths and weaknesses of the model. This can be done to gain a better understanding of how machine learning works. Evaluation of a model is required for determining its usefulness of a model during the preliminary stages of research. Evaluation of models also has a role to play in the monitoring of models. In this research, we make use of a wide variety of performance metrics, including,

- Peak Signal-to-Noise Ratio (PSNR)

The PSNR block determines the peak signal-to-noise ratio between two images and expresses the result in decibels. When comparing the quality of the original image to that of a compressed version, this ratio is the metric of choice. When the PSNR is higher, the quality of the compressed image, as well as the reconstructed image, is improved. Equation (14) is used to calculate the PSNR:

(14) $$PSNR=20log_{10}\left(\frac{MAX_{f}}{\sqrt{MSE}}\right)\;$$

Mean Square Error (MSE)

The MSE value denotes the average difference in all of the image’s pixels across the board. A greater value for MSE indicates a greater disparity between the original image and the image that has been processed. A mathematical presentation of MSE is found in Equation (15):

(15) $$\mathrm{MSE}=\frac{1}{\mathrm{mn}}\sum_{0}^{m-1}\sum_{0}^{n-1}∥f\left(i,j\right)-g(i,j){∥}^{2}$$

Structural Similarity Index (SSIM)

When comparing images, the Mean Squared Error, also known as MSE, which is simple to calculate, may not be a much better sign of how similar the images are perceived to be to one another. The Structural Similarity Index, also known as SSIM, is an attempt to make up for this deficiency. It does so by taking into account texture and awarding a higher score to images that may give the impression of being similar to one another. It is defined in Equation (16):

(16) $$\mathrm{SSIM}\left(x,y\right)=\frac{\left(2\mu_{x}\mu_{y}+c_{1}\right)\left(2\sigma_{\mathrm{xy}}+c_{2}\right)}{\left(\mu_{x}^{2}+\mu_{y}^{2}+c_{1}\right)\left(\sigma_{x}^{2}+\sigma_{y}^{2}+c_{2}\right)}$$

where

$\mu_{x}$ = is the average of x

$\mu_{y}$, = is the average of y

$\sigma_{x}^{2}$, = is the variance of x

$\sigma_{y}^{2}$, = is the variance of y

$\sigma_{\mathrm{xy}}$, = is the variance of x and xy

$c_{1}=\;\mathrm{represents}\;{\left(k_{1}L\right)}^{2}$

$c_{2}=\mathrm{represents}\;{\left(k_{2}L\right)}^{2}$

- Universal Image Quality Index (UQI)

The Universal Image Quality Index is first presented (UQI). Loss of correlation, luminance distortion, and contrast distortion are the three factors that are considered when determining the quality of an image by UQI. It is represented in Equation (17):

(17) $$\mathrm{UQI}=\frac{4\sigma_{\mathrm{xy}}\;\bar{x}\bar{y}}{\left(\sigma_{x}^{2}+\sigma_{y}^{2}\right)\left[{\left(\bar{x}\right)}^{2}+{\left(\bar{y}\right)}^{2}\right]}$$

- Accuracy

Accuracy can be defined as the proportion of total data points that correspond to accurate predictions. Formally, it is defined as the number of true positives and true negatives divided by the number of true positives, true negatives, false positives, and false negatives. It is represented in Equation (18):

(18) $$\mathrm{Accuracy}=\frac{\mathrm{True}\;\mathrm{Positive}+\mathrm{True}\;\mathrm{Negative}}{\mathrm{True}\;\mathrm{Positive}+\mathrm{False}\;\mathrm{Positive}+\mathrm{True}\;\mathrm{Negative}+\mathrm{False}\;\mathrm{Negative}\;}$$

- Precision

This refers to the quantification of the number of predictions for the positive class that belong to the positive class. It is represented in Equation (19):

(19) $$\mathrm{Precision}=\frac{\mathrm{True}\;\mathrm{Positive}}{\mathrm{True}\;\mathrm{Positive}+\mathrm{False}\;\mathrm{Positive}}$$

- Recall

This determines how many positive class predictions are made using all of the positive examples in the dataset. It is represented in Equation (20):

(20) $$\mathrm{Recall}=\frac{\mathrm{True}\;\mathrm{Positive}}{\mathrm{True}\;\mathrm{Positive}+\mathrm{False}\;\mathrm{Positive}}$$

- F1-score

This is an elegant way to summarize the predictive performance of a model by combining two metrics, precision and recall, that would otherwise compete with one another. It is represented in Equation (21):

(21) $$F1-\mathrm{score}=2\times \frac{\mathrm{Precision}\;\times \mathrm{Recall}}{\mathrm{Precision}\;+\mathrm{Recall}}$$

- Computational cost

The computational cost of a specific method or approach in machine learning or deep learning is simply a measurement of the number of resources that it uses. Knowing the computational cost is important so that we can determine how much time or computing power we require to train the model.

- Memory Utilization

Memory utilization is the average utilization that is derived from the percentage of available memory that is being used at any given moment. This percentage can be used as a starting point for calculating memory utilization.

## 4. Experiment, Result, and Findings

In Table 3, the specification of the hardware that is utilized in the experiment is outlined. For the experiment, a programming interface based on Python is used. We make use of a number of Python libraries, including NumPy, Pandas, Matplotlib, cv2, PyWavelets, Scikitplot, Sklearn, Scipy, and Skimage.

The experiments are carried out with great care and attention to detail by fine-tuning the various parameters. Converting the binary image of the malware into a grayscale image with dimensions of 224 by 224 is accomplished with the help of a Python script. It takes two days to complete the process of transforming binary data into an image. The initial size of the images was inconsistent, and this is because multiple malicious processes were running in the system before the memory acquisition step. However, in this paper, we keep the dimensions constant to reduce the amount of memory and computational cost. We believe that these images may contain different types of noise during the time of transformation or encoding. However, it is unknown what type of noise these images may contain. To denoise the images, we first added noise, such as Gaussian, salt-and-pepper, poisson, and speckle, to the original malware image to determine the filter techniques that can reduce or eliminate unwanted artifacts. Various filter techniques, such as CLAHE, bilateral filter, Wiener filter, Gaussian filter, non-local means, and total variation filter, are used. Each filter technique is extensively utilized with various values with respective parameters.

During the CLAHE denoising technique, it is observed that increasing the ‘tileGridSize’ parameter’s values resulted in a decrease in the performance evaluation, including MSE, PSNR, and UQI. The tileGridSize parameter divides the image into equal-sized rectangular tiles. The findings of the detailed experiments are presented in Table 4, which also reveals that the best performance was achieved by setting the ‘tileGridSize’ parameter to (1,1) and the ‘clipLimit’ parameter to 0.001, 0.01, respectively.

The bilateral filtering or bilateral smoothing technique introduces another Gaussian filter that takes into account the varying intensities to keep the edges intact. A bilateral filter denoising technique has a couple of important parameters, such as ‘diameter’, ‘sigmacolor’, and ‘sigmaspace’, to remove the noise from the image. We have tried a variety of different values for these parameters to determine the difference in effectiveness between the noisy and denoised images. Detailed experimentation of various parameter values is presented in Table 5. Here the dimension parameter is proportional to sigmaspace. The values 65, 5, and 50 for dimension, sigmacolor, and sigmaspace, respectively, produce the best results in terms of MSE, PSNR, and UQI.

Total variation with a Chambolle filter utilizes two crucial parameters, ‘noise’ and ‘weight’, to denoise the image. However, we found that the parameter with the name ‘eps’ has almost no impact on the deployment process. As a result, we constant its value to 0.002. The best possible outcome is accomplished through the utilization of this technique by setting the ‘weight’ value to 0.1. Table 6 presents comprehensive experimentation by making use of a variety of different values for its parameter.

The Wiener filter denoising technique is utilized in this experiment. It possesses two essential parameters, including ‘mysize’ and ‘noise’. Nonetheless, during deployment, we observed that the ‘mysize’ parameter has a significant impact on image denoising, whereas the ‘noise’ parameter makes little difference. Therefore, its values are used as a constant. The optimal result obtained with the ‘mysize’parameter was the value (3,3). Table 7 displays the results obtained by varying its parameter values.

A technique known as NL mean denoising is frequently employed to reduce the number of computations necessary. Patch size, h values, and patch distance are the three most important parameters to take into consideration. We have experimented extensively with the combination of a wide range of values to locate the result that provides the best PSNR, MSE, and UQI performance metrics. The degree of filtering that is applied is determined by the value of the parameter h, which takes the value 3, 2, or 1. Similarly, the values (11,9,5) and (3,5,7) are used for the parameter’s patch size and patch distance, respectively. We obtained the best possible result by setting the values for h, patch size, and patch distance to 1, 5, and 3, respectively. Table 8 displays the information in greater detail by making use of a combination of several different parameter values.

**Figure 10:**
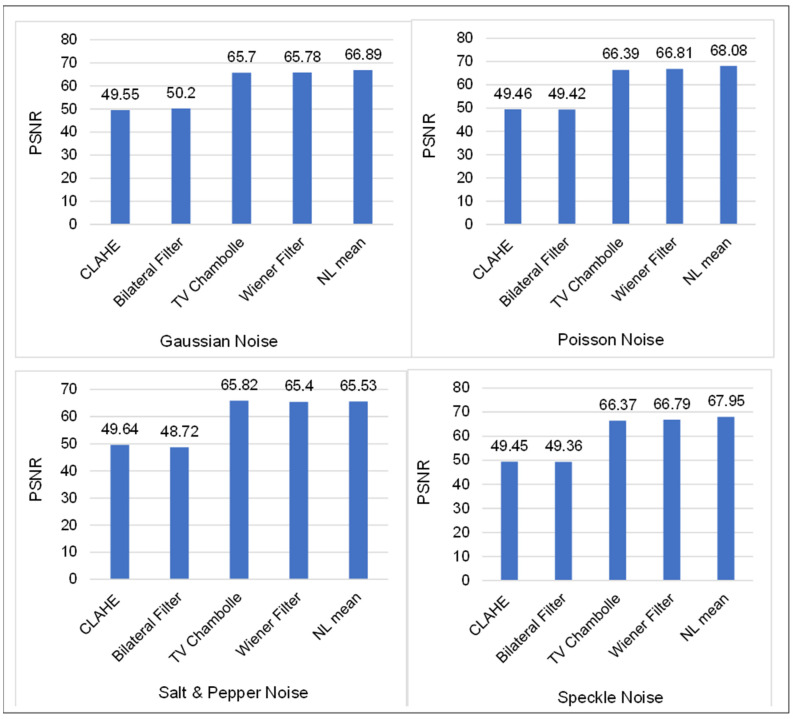
A comparison of denoising techniques in terms of PSN result for various images with noising.

The techniques that are used for denoising are evaluated alongside noise, such as Gaussian, salt-and-pepper, spackle, and poisson, to identify the most effective method and the values that should be assigned to their parameters. During the process of experimentation, we do not just use these denoising techniques singly; rather, we combine them to get the best possible result. Despite this, the observation reveals the integration negative impact on performance in terms of MSE, PSNR, and UQI. Figure 10 presents a comparison of the various denoising techniques applied to a malware image that has been affected by Gaussian, salt-and-pepper, speckle, and poisson noise. It demonstrates how the non-local means denoising technique can effectively remove noise without discarding an excessive amount of information.

At this point in the experimentation process, we have obtained an optimal technique for removing noise from malware images and are required to implement it on the original grayscale images dataset. After the implementation, the obtained denoised malware images dataset is fed into the wavelet transform to lower the memory utilization and computational cost. A DWT of wavelet transform techniques is utilized with level three for the compression, and, as a result, the dimensionality of the images is produced to the size of 28 × 28. Various families, such as Daubechies, Symlets, Biorthogonal, Coiflets, and Reverse Biorthogonal, are utilized during the experiment, and the results are evaluated using PSNR. PSNR values of 90.36 are produced by Daubechies, Biorthogonal, and Reverse Biorthogonal. Table 9 displays the various results obtained after deploying a variety of DWT families, along with the parameters that are specific to each family.

**Figure 11:**
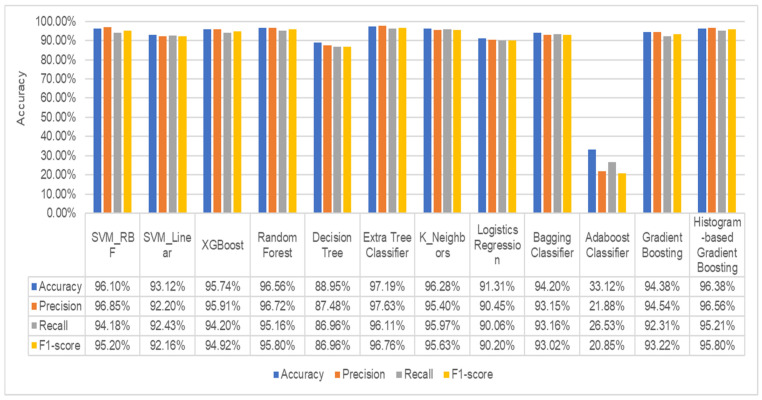
Ten widely used machine learning classifier results.

As a result, multiple families produced the same values of PSNR to evaluate the malware images. We have chosen to conduct our experiment using the Daubechies family’s db1 value. In the final step, the dataset of malware images is now being fed into the machine learning system to detect and classify malware families. We compared the accuracy, precision, recall, and f1-score of ten of the most popular machine learning classifiers. Initially, the malware image dataset is converted into a feature vector before being fed to classifiers. None of the machine learning classifiers’ parameters is tuned at the start of the process. The goal is to achieve the top five results, after which it is necessary to fine-tune its parameters to achieve further improvements. Figure 11 displays the experiment results using a variety of machine learning classifiers. The results show that the extra tree classifier performs well, with an accuracy of 97.19%, precision of 97.63%, recall of 96.11%, and f1-score of 96.76%. Machine learning techniques such as SVM with RBF kernel, histogram-based gradient boosting, k-nearest neighbor, and random forest are used to obtain the other closest results.

These top five classifiers have undergone hyperparameter tuning to improve overall performance. Table 10 provides an in-depth look at the hyperparameter settings for the top five machine learning classifiers, each of which is presented with its corresponding value. The SVC with kernel values of RBF, gamma 0.01, and C 100 shows the improvement in the results in terms of accuracy, precision, recall, and f1-score. Parameter C performs a crucial function by requiring the classifier to avoid misclassification and overfitting during training, whereas the gamma indicates the influence, with lower values indicating that the training data are distant, and higher values indicating that the training data are close.

**Figure 12:**
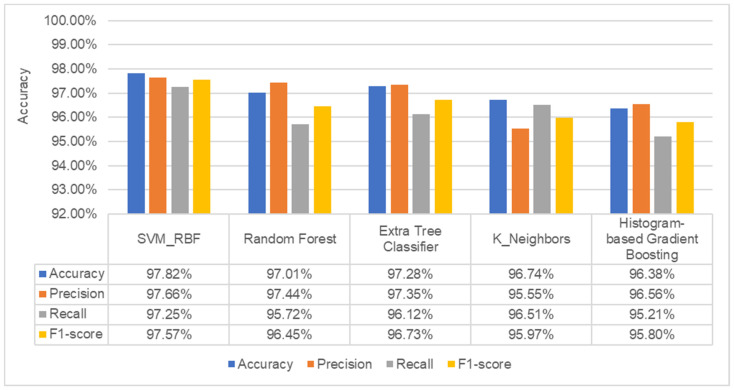
Results of best five machine learning classifiers after the hyper-tuning process.

After fine-tuning the parameters of the top five machine learning classifiers, we revealed that SVM with RBF kernel achieved the highest accuracy of 97.82%, precision of 97.66%, recall of 97.25%, and f1-score of 97.57%, while the extra tree classifier achieved the second highest accuracy of 97.28%, precision of 97.35%, recall of 96.12%, and f1-score of 96.73%. It is noted that hyper-tuning of SVM with RBF kernel improved its performance more than other classifiers. Figure 12 depicts the results obtained after hyperparameter tuning is performed.

**Figure 13:**
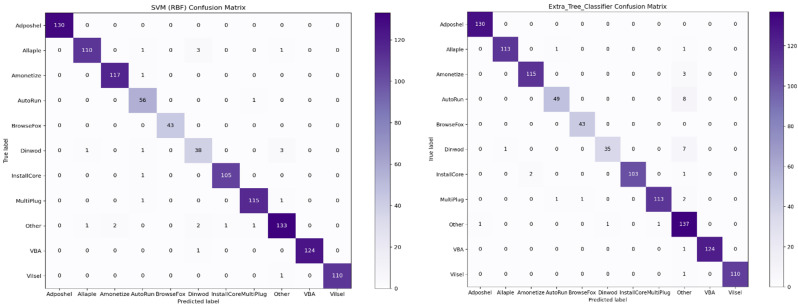

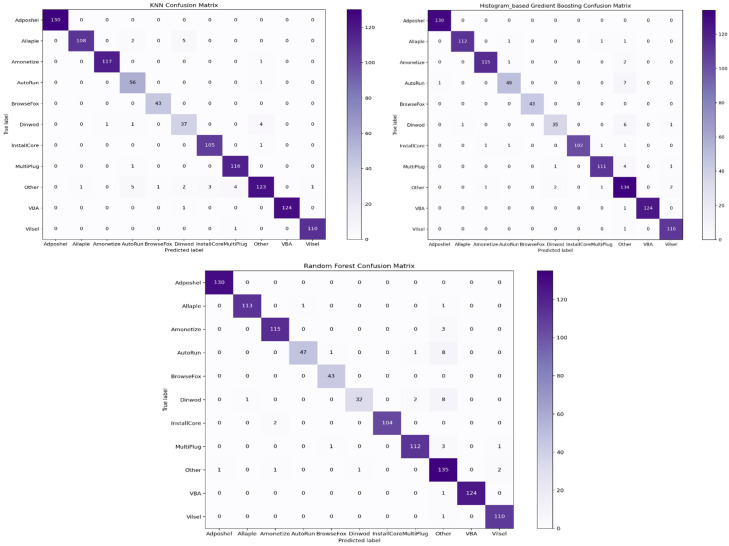
Confusion matrix of best five machine learning classifiers.

It is necessary to examine the confusion matrix of these classifiers to determine the prediction and types of errors, such as type-1 and type-II, to distinguish between various malware classes. The confusion matrix consists of two crucial dimensions: the actual label and the predicted label. Actual labels are the true values for the given observations, while predicted labels are the values predicted by the classifier. The confusion matrix of the top five high-performance classifiers is depicted in Figure 13. The ‘Dinwod’ malware class performed the worst among all classifiers, and this is due to its visual similarity to the benign “other” class. Similarly, the ‘Adposhel’ and ‘BrowseFox’ classes are classified with 100% accuracy by all classifiers. It is noted that these classes are dissimilar, making it simple for classifiers to differentiate between them. In terms of binary classification, the confusion matrix also reveals that KNN and SVM with an RBF kernel are confused between benign and malicious images, despite the fact that both classifiers accurately identified various other malware classes.

**Figure 14:**
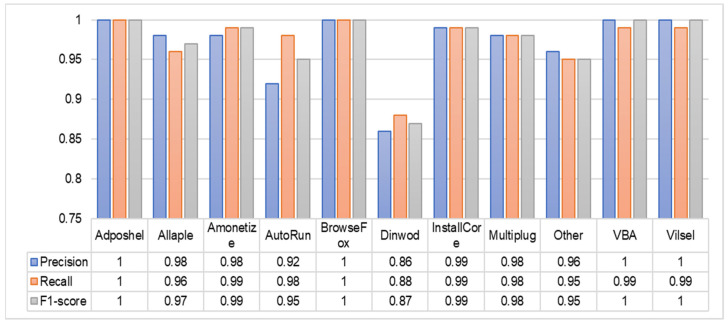
Classification report of SVM with RBF kernel.

To calculate the precision, recall, and f1-score for each class of malware, we display the classification of various malware classes in tabular format for the SVM with RBF kernel. Figure 14 displays the information in greater detail regarding the classification report generated by SVM with RBF kernel for each class in terms of precision, recall, and f1-score. These metrics of performance, as well as accuracy, must be given equal consideration. According to the literature review, accuracy does not always provide the most effective result; as a result, other performance metrics should be considered. The results demonstrate that malware classes such as ‘Adposhel’, ‘BrowseFox’, and ‘VBA’ performed exceptionally well using the SVM with RBF kernel classifier of machine learning, whereas ‘Dinwod’ performed the worst among the remaining classes. There could be a number of reasons why this class of malware performs less well than others, including (i) the lack of insufficient training data for the classifier, (ii) the loss of a large number of sensitive data during compression, and (iii) insufficient noise removed by denoising techniques. To determine the root cause, additional investigation is still necessary. In conclusion, our techniques improve not only accuracy, but also precision, recall, f1-score, and memory storage, while reducing the computational cost.

To demonstrate the effectiveness of the proposed techniques in detecting and classifying malware classes when applied to an unbalanced dataset, it is observed that the performance of the proposed technique outperformed other relevant approaches based on visualization. We compared the outcome to the most recent research published in [12,13,36,38,59]. The experimentation shows that feature engineering is a critical step before the data is fed into machine learning or deep learning. Feature engineering techniques such as GIST descriptor combined with machine learning are proposed in [12]. Similarly, a HOG feature descriptor in conjunction with multi-layer perceptron is proposed in [38]. A combination of the positive features of both these feature descriptors is proposed in [13]. However, it is observed that it is computationally and memory-intensive. To circumvent the high computational cost and memory consumption, our proposed method effectively reduces the cost in terms of computing and memory and outperforms the competition in terms of accuracy, precision, recall, and f1-score. Our proposed technique begins by denoising the images to eliminate irrelevant texture features, and then it reduces the dimensions of these images to a size of 28 × 28 to maintain the essential texture features, while minimizing the amount of information that is lost. Table 11 compares the performance of the proposed technique to the most pertinent research.

## 5. Discussion

We used a denoising technique to remove noise from the converted malware images, which improved the performance of machine learning models in terms of accuracy, precision, recall, and f1-score. We considered common noise types, such as Gaussian, Salt-and-Pepper, Speckle, and Poisson, and applied various denoising filter techniques to determine the optimal values for each parameter. However, one of the unanticipated results demonstrates that a malware class called ‘Dinwod’ performed poorly, indicating that the denoising technique removed too many important artifacts from the images and made it difficult for the machine learning model to differentiate between various malware types. It is therefore necessary to conduct additional research into the cause of the loss of crucial malware artifacts belonging to specific classes.

In this paper, we used DWT compression techniques to reduce the dimensionality and remove the irrelevant or duplicate artifacts from malware images without sacrificing too many key artifacts. Using this technique yields results that are highly efficient in terms of computation and memory consumption. Nonetheless, throughout the experimental phase, we discovered that the performance of distinct malware classes changes with DWT levels 1, 2, and 3. The findings reveal that the procedure of dimensionality reduction and compression performed well for the majority of malware classes, but degraded the performance for a few forms of malware, necessitating more investigation. Lastly, in this paper, we do not consider a feature selection technique to further reduce the number of features. We are hopeful that, by utilizing a feature selection technique, the cost of computing and memory usage can be further reduced without sacrificing performance accuracy.

## 6. Conclusions

The detection and classification of malware play a crucial role in cyber security. It is necessary to comprehend the behavior of malware to detect it. The most prevalent analysis methods include static, dynamic, and hybrid. However, such an analysis requires domain-specific expertise to identify the crucial characteristics of a suspicious file, which is not always possible. In this paper, memory dump files from malware are converted into grayscale images to avoid necessitating domain expertise. We believed that during the dumping and visualization processes, image noise is also introduced. Numerous studies have proposed detecting and classifying malware using visualization. However, none of the researchers emphasizes the significance of denoising visual images. This paper proposes two important techniques: non-local means denoising for noise removal and discrete wavelet transform for image compression to reduce image dimensions. Finally, these images are fed into classifiers that utilize machine learning. SVM with RBF kernel achieved the best accuracy, precision, recall, and f1-score among the best classifiers. In the future, it will be necessary to focus on malware classes for which the detection rate is less than 90%. The denoising of grayscale malware images is still novel in the detection and classification of malware, and we are optimistic that more research is necessary. This study has not evaluated cloud computing technology; nonetheless, we are hopeful that using such an approach will also improve the level of cybersecurity in an online environment.

## Figures and Tables

**Table 1:** Memory-based malware dataset.

Category	Classes	Quantity
Adware	Adposhel	457
Worm	Allaple.A	437
Adware	Amonetize	436
Worm	AutoRun-PU	196
Adware	BrowseFox	190
Trojan	Dinwod!rfn	127
Adware	InstallCore.C	467
Adware	MultiPlug	488
Trojan	Vilsel	389
Virus	VBA	499
Benign	--	608

**Table 3:** Hardware Specifications.

Parameters	Values
Operating System	Windows 7 Professional—64 bit
RAM	32
Processor	2.40 GHz
Hard Drive	512 SSD

**Table 4:** Implementation of the CLAHE technique with diverse parameter values.

Noise Type	CLAHE Parameters		Evaluation Metrics			
	tileGridSize	clipLimit	MSE	PSNR	SSIM	UQI
Gaussian	(1,1)	0.001	0.7203	49.55	0.9996, 0.9999	0.9994
		0.01	0.7203	49.55	0.9996, 0.9999	0.9994
		0.05	0.7251	49.52	0.9996, 0.9996	0.9994
		0.1	0.9854	48.19	0.9996, 0.9996	0.9993
		0.2	3.4	42.8	0.9994, 0.9999	0.9991
		0.5	59.93	30.35	0.9994, 0.9981	0.9937
	(2,2)	0.001	0.732	49.48	0.9996, 0.9996	0.9993
		0.01	0.732	49.48	0.9996, 0.9999	0.9993
		0.05	0.7673	49.28	0.9996, 0.9999	0.9993
		0.1	0.9815	48.21	0.9996, 0.9999	0.9993
		0.2	3.9	42.21	0.9992, 0.9999	0.9989
		0.5	56.26	30.62	0.9946, 0.9981	0.9935
Salt-and-Pepper	(1,1)	0.001	0.7063	49.64	0.99924, 0.9999	0.9924
		0.01	0.7063	49.64	0.99924, 0.9999	0.9924
		0.05	0.8246	48.96	0.9992, 0.9999	0.9923
		0.1	0.973	48.24	0.9992, 0.9999	0.9923
		0.2	5.08	41.07	0.9998, 0.9999	0.9919
		0.5	48.52	31.27	0.9995, 0.9984	0.9886
	(2,2)	0.001	0.8209	48.98	0.9992, 0.9999	0.9923
		0.01	0.8209	48.98	0.9992, 0.9999	0.9923
		0.05	0.9708	48.25	0.9992, 0.9999	0.9923
		0.1	2.6	43.96	0.9991, 0.9999	0.9922
		0.2	5.95	40.38	0.9988, 0.9998	0.9918
		0.5	47.87	31.32	0.99954, 0.9983	0.9884
Poisson	(1,1)	0.001	0.7347	49.46	0.9951, 0.9999	0.9559
		0.01	0.7347	49.46	0.9951, 0.9999	0.9559
		0.05	0.7873	49.16	0.9951, 0.9999	0.9559
		0.1	0.9971	48.14	0.9951, 0.9999	0.9559
		0.2	5.44	40.77	0.9947, 0.9998	0.9554
		0.5	70.63	29.64	0.9893, 0.9977	0.9495
	(2,2)	0.001	0.7755	49.23	0.9951, 0.9999	0.9559
		0.01	0.7755	49.23	0.9951, 0.9999	0.9559
		0.05	0.8904	48.63	0.9951, 0.9999	0.9559
		0.1	0.9954	48.15	0.9951, 0.9999	0.9559
		0.2	6.63	39.91	0.9946, 0.998	0.9552
		0.5	65.25	29.98	0.9893, 0.9976	0.9497
Speckle	(1,1)	0.001	0.737	49.45	0.9951, 0.9999	0.956
		0.01	0.737	49.45	0.9951, 0.9999	0.956
		0.05	0.7849	49.18	0.9951, 0.9999	0.956
		0.1	0.9956	48.14	0.9951, 0.9999	0.9559
		0.2	4.64	41.46	0.9948, 0.9999	0.9555
		0.5	69.83	29.68	0.9894, 0.9977	0.9497
	(2,2)	0.001	0.7608	49.31	0.9951, 0.9999	0.956
		0.01	0.7608	49.31	0.9951, 0.9999	0.956
		0.05	0.84	48.88	0.9951, 0.9999	0.956
		0.1	0.9947	48.15	0.9951, 0.9999	0.9559
		0.2	6.58	39.94	0.9946, 0.9998	0.9553
		0.5	66.35	29.91	0.9892, 0.9975	0.9496

**Table 5:** Implementation of the bilateral filter with diverse parameter values.

Noise Type	Bilateral Filter Parameters			Evaluation Metrics		
	Dimension	SigmaColor	SigmaSpace	MSE	PSNR	UQI
Gaussian	5	35	10	167.54	25.88	0.9674
			50	168.02	25.87	0.9676
			150	169.54	25.83	0.9686
		50	10	370.48	22.42	0.9489
			50	372.8	22.41	0.9492
			150	373.33	22.4	0.9489
	25	35	10	199.57	25.12	0.9603
			50	202.14	25.07	0.9595
			150	203.41	25.04	0.9597
		50	10	480.9	21.31	0.9331
			50	484.81	21.27	0.9316
			150	487.43	21.25	0.9313
	55	5	10	0.7149	49.58	0.9996
			15	0.9597	49.93	0.9997
			150	0.6559	49.96	0.9997
		15	10	15.19	36.31	0.9942
			50	15.35	36.26	0.9942
			150	15.28	36.28	0.9942
	65	5	10	0.7075	49.63	0.9996
			50	0.62	50.2	0.9997
			150	0.6314	50.09	0.9996
		15	10	15.31	36.28	0.9938
			50	14.96	36.38	0.9944
			150	15.05	36.35	0.9943
Salt-and-Pepper	5	35	10	141.64	26.61	0.9872
			50	142.28	26.59	0.9884
			150	142.19	26.6	0.9867
		50	10	324.8	23.01	0.979
			50	325.15	23	0.9785
			150	326.47	22.99	0.9784
	25	35	10	186.26	25.42	0.9747
			50	187.44	25.4	0.974
			150	189.34	25.35	0.9683
		50	10	475.98	21.35	0.9543
			50	482.74	21.29	0.9501
			150	481.88	21.3	0.9526
	55	5	10	0.8669	48.75	0.9997
			15	0.8613	48.77	0.9996
			150	0.8903	48.63	0.9995
		15	10	13.91	36.69	0.9907
			50	13.86	36.71	0.9919
			150	13.92	36.69	0.9874
	65	5	10	0.8757	48.7	0.9996
			50	0.8722	48.72	0.9997
			150	0.8817	48.67	0.9997
		15	10	13.65	36.77	0.991
			50	13.56	36.8	0.9901
			150	13.51	36.82	0.9888
Poisson	5	35	10	147.79	26.43	0.967
			50	148.47	26.41	0.967
			150	148.5	26.41	0.967
		50	10	325.2	23	0.9511
			50	326.27	22.99	0.951
			150	326.32	22.99	0.951
	25	35	10	193.73	26.25	0.9224
			50	167.22	25.18	0.9209
			150	197.36	25.17	0.9203
		50	10	464.03	21.46	0.8926
			50	471.63	21.39	0.8907
			150	471.92	21.39	0.8906
	55	5	10	0.7635	49.3	0.999
			15	0.755	49.35	0.999
			150	0.7562	49.34	0.999
		15	10	14.82	36.42	0.9522
			50	15.21	36.3	0.9519
			150	15.23	36.3	0.9519
	65	5	10	0.7771	49.22	0.9988
			50	0.742	49.42	0.9988
			150	0.7439	49.41	0.9988
		15	10	14.87	36.4	0.9523
			50	14.96	36.37	0.952
			150	14.98	36.37	0.952
Speckle	5	35	10	147.73	26.43	0.9667
			50	148.39	26.41	0.9667
			150	148.41	26.41	0.9667
		50	10	325.33	23	0.9512
			50	326.48	22.99	0.9511
			150	326.53	22.99	0.9511
	25	35	10	196.71	25.19	0.9226
			50	200.32	25.11	0.9213
			150	200.42	25.11	0.9212
		50	10	471.37	21.39	0.8928
			50	479.16	21.32	0.8909
			150	479.43	21.32	0.8909
	55	5	10	0.7767	49.22	0.9987
			15	0.7722	49.25	0.9987
			150	0.7756	49.23	0.9987
		15	10	15.05	36.35	0.9522
			50	15.38	36.25	0.9519
			150	15.4	36.25	0.9519
	65	5	10	0.7925	49.14	0.9989
			50	0.7527	49.36	0.9989
			150	0.7523	49.36	0.9989
		15	10	14.9	36.39	0.9522
			50	14.95	36.38	0.9519
			150	14.19	36.37	0.9519

**Table 6:** Implementation of the total variation—Chambolle with diverse parameter values.

Noise Type	TV Chambolle Parameters		Evaluation Metrics		
	Weight	eps	MSE	PSNR	UQI
Gaussian	**0.1**	**0.0002**	**0.0174**	**65.7**	**0.8986**
	0.2	0.0002	0.0299	63.37	0.8409
	0.3	0.0002	0.03	63.35	0.84
	0.4	0.0002	0.034	62.8	0.8193
	0.5	0.0002	0.0358	62.8	0.8105
	0.6	0.0002	0.37	62.44	0.805
	0.7	0.0002	0.0377	62.35	0.0801
	0.8	0.0002	0.0382	62.3	0.7989
	0.9	0.0002	0.0385	62.27	0.7971
Salt-and-Pepper	0.1	0.0002	0.017	65.82	0.9282
	0.2	0.0002	0.0337	62.84	0.833
	0.3	0.0002	0.038	62.33	0.7879
	0.4	0.0002	0.0406	62.04	0.7692
	0.5	0.0002	0.0429	61.79	0.756
	0.6	0.0002	0.0443	61.66	0.7488
	0.7	0.0002	0.0452	61.57	0.7441
	0.8	0.0002	0.0457	61.52	0.7415
	0.9	0.0002	0.0461	61.48	0.7395
Speckle	0.1	0.0002	0.0149	66.37	0.8784
	0.2	0.0002	0.0254	64.07	0.8132
	0.3	0.0002	0.0256	64.04	0.814
	0.4	0.0002	0.0294	63.44	0.787
	0.5	0.0002	0.0312	63.18	0.7751
	0.6	0.0002	0.0326	62.99	0.766
	0.7	0.0002	0.0333	62.89	0.7609
	0.8	0.0002	0.0338	62.84	0.7579
	0.9	0.0002	0.034	62.8	0.7562
Poisson	0.1	0.0002	0.0149	66.39	8777
	0.2	0.0002	0.0217	64.75	0.8394
	0.3	0.0002	0.0252	64.1	0.815
	0.4	0.0002	0.029	63.49	0.7888
	0.5	0.0002	0.0308	63.23	0.7761
	0.6	0.0002	0.0322	63.04	0.767
	0.7	0.0002	0.0329	62.94	0.7619
	0.8	0.0002	0.0334	62.88	0.7589
	0.9	0.0002	0.0336	62.85	0.7572

**Table 7:** Implementation of the Wiener filter with diverse parameter values.

Noise Type	Wiener Filter Parameters	Evaluation Metrics		
	Mysize	MSE	PSNR	UQI
Gaussian	(5,5)	0.0224	64.54	0.8669
	(7,7)	0..0260	63.96	0.8411
	(9,9)	0.0278	63.68	0.8316
	(11,11)	0.0291	63.48	0.8248
	(3,3)	0.0171	65.78	0.902
	(13,13)	0.0301	63.34	0.8192
Salt-and-Pepper	(5,5)	0.0279	63.66	0.841
	(7,7)	0.0323	63.02	0.8026
	(9,9)	0.0348	62.7	0.7835
	(11,11)	0.0364	62.51	0.7735
	(3,3)	0.0187	65.4	0.9147
	(13,13)	0.0376	62.37	0.765
Speckle	(5,5)	0.0187	65.39	0.8532
	(7,7)	0.022	64.69	0.8107
	(9,9)	0.0237	64.38	0.7943
	(11,11)	0.025	64.15	0.7879
	(3,3)	0.0136	66.79	0.8879
	(13,13)	0.0259	63.98	0.7804
Poisson	(5,5)	0.0186	65.42	8513
	(7,7)	0.0218	64.72	0.8118
	(9,9)	0.0234	64.42	0.7984
	(11,11)	0.0247	64.19	0.7884
	(3,3)	0.0135	66.81	0.8872
	(13,13)	0.0256	64.03	0.7811

**Table 8:** Implementation of the non-local means with diverse parameter values.

Noise Type	Non Local Means Parameters			Evaluation Matrices		
	Patch Size	h Values	Patch Distance	MSE	PSNR	UQI
Gaussian	11	3	3	0.0351	62.67	0.8127
			5	0.0384	62.28	0.7957
			7	0.0394	62.17	0.789
		2	3	0.0322	63.04	0.8245
			5	0.0358	62.58	0.8045
			7	0.0373	62.4	0.7967
	9	3	3	0.0351	62.67	0.8123
			5	0.038	62.32	0.7957
			7	0.0388	62.23	0.7919
		2	3	0.0317	63.11	0.8253
			5	0.0353	62.64	0.8069
			7	0.0369	62.45	0.7965
	5	2	3	0.0302	63.31	0.8318
			5	0.0335	62.87	0.8121
			7	0.0344	62.76	0.8058
		1	3	0.0132	66.89	0.8989
			5	0.0189	65.35	0.8663
			7	0.0209	64.92	0.8555
Salt-and-Pepper	11	3	3	0.0351	62.67	0.8135
			5	0.0382	62.3	0.7957
			7	0.0391	62.19	0.7892
		2	3	0.0319	63.08	0.8251
			5	0.0357	62.59	0.8051
			7	0.0371	62.42	0.7969
	9	3	3	0.0425	61.84	0.7592
			5	0.0457	61.52	0.7402
			7	0.0466	61.44	0.7335
		2	3	0.0391	62.19	0.7734
			5	0.0432	61.77	0.7502
			7	0.0445	61.63	0.7416
	5	2	3	0.037	62.44	0.7813
			5	0.0405	62.04	0.759
			7	0.0417	61.92	0.7515
		1	3	0.0181	65.53	0.8836
			5	0.0235	64.4	0.8443
			7	0.026	63.97	0.8295
Poisson	11	3	3	0.0294	63.43	0.7862
			5	0.0327	62.97	0.76
			7	0.0339	62.82	0.7489
		2	3	0.0255	64.05	0.8022
			5	0.0294	63.44	0.7743
			7	0.031	63.21	0.7609
	9	3	3	0.0293	63.46	0.7872
			5	0.0326	62.99	0.7606
			7	0.0337	62.85	0.7498
		2	3	0.0253	64.08	0.8034
			5	0.0292	63.47	0.7744
			7	0.0307	63.25	0.7615
	5	2	3	0.0241	64.3	0.808
			5	0.0272	63.77	0.7814
			7	0.0283	63.6	0.7703
		1	3	0.0101	68.08	0.8811
			5	0.0135	66.82	0.8428
			7	0.015	66.36	0.8317
Speckle	11	3	3	0.0298	63.38	0.7435
			5	0.0331	62.92	0.7594
			7	0.0342	62.78	0.7483
		2	3	0.0259	63.98	0.7989
			5	0.0298	63.38	0.7733
			7	0.0314	63.15	0.76
	9	3	3	0.0297	63.4	0.7894
			5	0.033	62.94	0.7596
			7	0.0341	62.8	0.7486
		2	3	0.0258	64.01	0.7996
			5	0.0296	63.4	0.773
			7	0.0312	63.18	0.76
	5	2	3	0.0245	64.22	0.8085
			5	0.0277	63.7	0.7801
			7	0.0288	63.52	0.769
		1	3	0.0104	67.95	0.878
			5	0.0138	66.7	0.8404
			7	0.0154	66.24	0.829

**Table 9:** Discrete Wavelet Transform implementation with diverse families.

Family	Discrete Wavelet Transform Parameters	Evaluation Metric
		PSNR
Daubechies	db1	90.36
	db2	95.17
	db3	49.55
	db4	56.35
	db5	54.1
	db6	50.02
	db7	49.45
	db8	50.22
	db9	50.83
	db10	53.01
Symlets	sym2	51.14
	sym3	51.05
	sym4	51.07
	sym5	51.34
	sym6	51.14
	sym7	51.26
	sym8	51.17
	sym9	48.63
Biorthogonal	bior1.1	90.36
	bior1.3	75.79
	bior1.5	70.15
	bior2.2	56.7
	bior2.4	68.45
	bior2.6	64.17
	bior2.8	66.44
	bior3.1	48.13
	bior3.3	54.63
	bior3.5	54.01
	bior3.7	53.72
	bior3.9	53.7
	bior4.4	51.17
	bior5.5	5.09
	bior6.8	51.14
Coiflets	coif1	57.7
	coif2	58.24
	coif3	56.31
	coif4	50.89
	coif5	52.54
	coif6	56.15
	coif7	57.62
	coif8	58.37
	coif9	51.51
	coif10	54.15
Reverse Biorthogonal	rbior1.1	90.36
	rbior1.3	71.75
	rbior1.5	69.87
	rbior2.2	52.85
	rbior2.4	57.96
	rbior2.6	57.23
	rbior2.8	59.82
	rbior3.1	49.38
	rbior3.3	51.65
	rbior3.5	51.96
	rbior3.7	52.15
	rbior3.9	52.37
	rbior4.4	51.17
	rbior5.5	51.09
	rbior6.8	51.14

**Table 10:** Hyper-tuning process of top five machine learning classifiers.

Classifiers	Parameters Name	Parameters Values	Optimal Values
SVC	C	1, 5, 10, 100, 1000	100
	gamma	1, 0.1, 0.01, 0.001, 0.0001	0.01
	kernel	‘RBF’, ‘Linear’	‘RBF’
Extra Tree Classifier	n_estimators	10, 50, 100, 200, 300	300
	criterion	‘gini’, ‘entropy’, ‘log_loss’	‘Gini’
Histogram-based Gredient Boosting	learning_rate	0.1, 0.01, 0.001, 0.0001	0.1
	loss	‘log_loss’, ‘auto’,‘categorical_crossentropy’	‘categorical_crossentropy’
	l2_regularization	0, 1	0
K_Nearest Neighbor	n_neighbors	1, 5, 10, 15, 20	1
	weights	‘uniform’, ‘distance’	‘uniform’
	algorith	‘auto’, ‘ball_tree’, ‘kd_tree’, ‘brute’	‘auto’
Random Forest	n_estimators	10, 50, 100, 155, 200	50
	criterion	‘gini’, ‘entropy’, ‘log_loss’	‘gini’

**Table 11:** A comparison of achieved results with the most relevant research.

S.No	Ref.	Accuracy	Precision	Recall	F1-Score
1	[12]	91.40%	91.50%	91.40%	91.50%
2	[38]	94.54%	94.60%	94.50%	94.50%
3	[59]	96.93%	97.00%	96.90%	96.90%
4	[13]	96.36%	96.40%	96.40%	96.40%
5	[36]	97.01%	97.36%	95.65%	96.36%
6	Proposed	97.82%	97.66%	97.25%	97.57%

## Data Availability

Not applicable.
